# The Underestimated Threat—Mycobacterium Genavense Infection: A Case Report

**DOI:** 10.3390/idr17030060

**Published:** 2025-06-01

**Authors:** Jannik Sonnenberg, Gert Gabriels, Ioana Diana Olaru, Sebastian Mühl, Julia Fischer, Hermann Pavenstädt, Jonel Trebicka, Kai-Henrik Peiffer, Phil-Robin Tepasse

**Affiliations:** 1 Department of Medicine B for Gastroenterology, Hepatology, Endocrinology and Clinical Infectiology, University Hospital Münster, 48149 Münster, Germany; 2 Department of Medicine D for General Internal Medicine, Nephrology and Rheumatology, University Hospital Münster, 48149 Münster, Germany; 3 Institute of Medical Microbiology, University Hospital Münster, 48149 Münster, Germany; 4 Gerhard Domagk Institute of Pathology, University Hospital Münster, 48149 Münster, Germany

**Keywords:** nontuberculous mycobacteria, fever of unknown origin, pancytopenia, bone marrow, case report

## Abstract

Background/Objectives: Nontuberculous mycobacteria (NTM) represent a heterogeneous group of pathogens with increasing global prevalence and significant geographical variation in species distribution. NTM infections, often affecting immunocompromised individuals, are difficult to diagnose due to nonspecific clinical presentations and laboratory findings. This case study presents a rare extrapulmonary NTM infection in a 73-year-old man, initially misdiagnosed as sarcoidosis, highlighting the diagnostic and therapeutic challenges posed by such infections. Methods: The patient, a pigeon fancier, presented with recurrent fever and pancytopenia. Extensive diagnostics included blood cultures, bone marrow aspiration, and histopathology. Initial cultures and serological tests remained negative. Results: Bone marrow aspiration revealed epithelioid granulomas, initially leading to the provisional diagnosis of sarcoidosis. However, after six weeks, *M. genavense* was isolated from mycobacterial blood cultures from bone marrow aspirant. Antimicrobial therapy with azithromycin, rifampicin, and ethambutol was initiated. Following the initiation of appropriate antimycobacterial therapy, the patient developed immune reconstitution inflammatory syndrome (IRIS), which was managed with supportive care. The patient’s condition improved, and no further febrile episodes occurred post-treatment, marking the successful conclusion of NTM therapy. Conclusions: This case underscores the diagnostic complexity of extrapulmonary NTM infections, particularly in immunocompromised patients. Misdiagnosis can delay appropriate treatment. *M. genavense*, though rare, should be considered in patients with a fever of unknown origin, especially with a background of immunosuppression. Prompt mycobacterial testing and tailored antibiotic therapy are crucial to improving outcomes in NTM infections.

## 1. Introduction

Nontuberculous mycobacterial (NTM) infections are characterized by extreme heterogeneity, affecting virtually any site from skin to cerebrospinal fluid. Epidemiologic studies of NTM disease show tremendous geographic variations in prevalence and mycobacterial species. Disseminated infections are mainly accompanied by fever followed by cytopenia, which can be found in less than 50% of patients [1].

NTM prevalence is higher in women and in Asia compared to Europe, Australia, or North America, though generally showing increasing prevalence throughout the study periods of existing epidemiological studies from 1999 to 2016 [2,3,4]. An analysis of German pulmonary NTM patients estimated a prevalence of 5.1 to 5.6 per 100,000 persons without changes in the 5-year study period from 2016 to 2020 [5]. This analysis did not include extrapulmonary samples, which comprised less than one-fourth of the total samples. The most frequent extrapulmonary involvements include disseminated infection, intraabdominal, central nervous system, skin, soft tissue, and bones [6,7]. The distribution of NTM pathogens is geographically specific. [8]. With mortality rates of up to 35% in patients requiring treatment, NTM infections represent a serious emerging health issue with a worldwide steady increase in incidence and prevalence [9].

*M. genavense* is an opportunistic pathogen with significant clinical implications, particularly in immunocompromised populations, including HIV/AIDS patients, solid-organ transplant recipients, patients with genetic immunodeficiencies, and those on long-term immunosuppressive therapies [10,11]. The main clinical findings include fever, weight loss, fatigue, enlarged lymph nodes, bone and joint pain, and skin lesions. In case of pulmonary involvement cough, increased sputum production, dyspnoea, and chest tightness may be present. Cytopenia and granulomatous inflammation of the afflicted site are laboratory and histological hallmarks of NTM diseases [12]. *M. genavense* infection is associated with high mortality if untreated, particularly in delayed diagnosis [13].

However, due to the unspecific and variably graded clinical manifestations and the possible similarity of the signs and symptoms of multiple diseases, recognition of *M. Genavense* infections can be difficult. Non-caseating granulomas in lymph nodes or tissues are histopathologically indistinguishable from sarcoidosis, increasing the risk of misdiagnosis and putting patients at risk of disease exacerbation under steroid treatment. In European hospitals, testing for NTM is not standard procedure, and rapid testing methods are not widely implemented [14,15].

Therapeutic options to treat NTM infection are dependent on the species, and resistance patterns differ between species and between slowly and rapidly growing mycobacteria. Due to the variability of species and lack of randomized trials, recommendations for the treatment of extrapulmonary manifestations of rare species are limited. This is aggravated further, as resistance testing in slow-growing NTM like *M. genavense* is not always possible, and in vitro susceptibility shows no significant correlation to the in vivo response in many organisms [16]. Therefore, therapeutic regimes usually consist of a combination of up to three antibiotics and should be continued for at least 12 months [17].

## 2. Materials and Methods

The information presented within this case report was collected throughout treatment at the University Hospital Münster, Germany, between April 2022 and September 2023.

## 3. Case Presentation

A 73-year-old male pensioner and pigeon fancier from Germany presented to our tertiary medical center with reoccurring fever, pronounced weakness, and pancytopenia. His medical history included rheumatoid arthritis, splenomegaly, cholecystolithiasis, diverticulosis, spondylodesis, recent myocardial infarction, and an aortic aneurysm. Rheumatoid arthritis was being treated with oral prednisolone 10 mg once daily and leflunomide 20 mg once daily, as well as methotrexate 20 mg injections once per week for the past years. Due to the recent myocardial infarction and stent implantation, double antiplatelet therapy with acetylsalicylic acid and prasugrel was in place. He was treated for infection and sepsis at secondary hospitals in the two months leading up to admission. Antibiotic therapy had previously been conducted with ampicillin/sulbactam, piperacillin/tazobactam, meropenem, azithromycin, ciprofloxacin, and doxycycline. Cytopenia had been attributed to disease-modifying anti-rheumatic drug therapy and treated with G-CSF injection. Upon admission he presented lower back pain, slight peripheral edema, and reduced vigilance. The patient showed hypotension without antihypertensive medication. The initial laboratory findings included pancytopenia (hemoglobin 8.1 g/dL, leucocytes 2.05 × 10^3^/µL, and thrombocytes 101 × 10^3^/µL), including lymphopenia (11.1%), signs of inflammation (CRP 3.2 mg/dL), elevated serum creatinine (1.22 mg/dL), and bilirubin (1.6 mg/dL).

For an overview of the patient’s baseline characteristics, see Table 1.

The laboratory results 5 months prior to the current episodes throughout treatment for myocardial infarction showed leucocytes and thrombocytes within normal ranges and chronic renal impairment. Examinations performed in external hospitals prior to transfer into our tertiary hospital included transesophageal echocardiography (TEE) to exclude endocarditis and endoscopic ultrasound (EUS) and endoscopic retrograde cholangiopancreatography (ERCP) due to increased liver enzymes, which yielded no significant results. Suspected spondylodiscitis could not be ruled out via MRI, as the examination had to be interrupted due to pain exacerbation suspected to stem from lumbosacral spondylodesis.

The laboratory diagnostics at our hospital yielded no significant results, and the initial blood cultures were negative. The workup further excluded viral hepatitis A/B/C, autoimmune hepatitis, HIV, EBV, CMV, HSV 1/2, *Salmonella* spp., *Yersinia enterocolitica*, *Campylobacter* spp., *Shigella* spp., *Mycoplasma pneumoniae*, *Chlamydia pneumoniae*, *Legionella*, SARS-CoV-2, hypercortisolism, and procalcitonin elevation. Felty syndrome was considered an unlikely differential diagnosis due to the lack of destructive rheumatoid arthritis and asymptomatic patient under treatment. Empirical piperacillin/tazobactam therapy resulted in no clinical or laboratory changes and was discontinued after three days. As extensive radiological (ultrasound, CT, and MRI) and invasive diagnostics (TEE, EUS, and ERCP) had already been performed externally, FDG-PET CT was planned, showing a possible focus in the central venous catheter previously established during intensive care unit treatment without further foci. Additionally, computer tomography revealed mediastinal and bilateral hilar lymphadenopathy.

**Figure 1:**
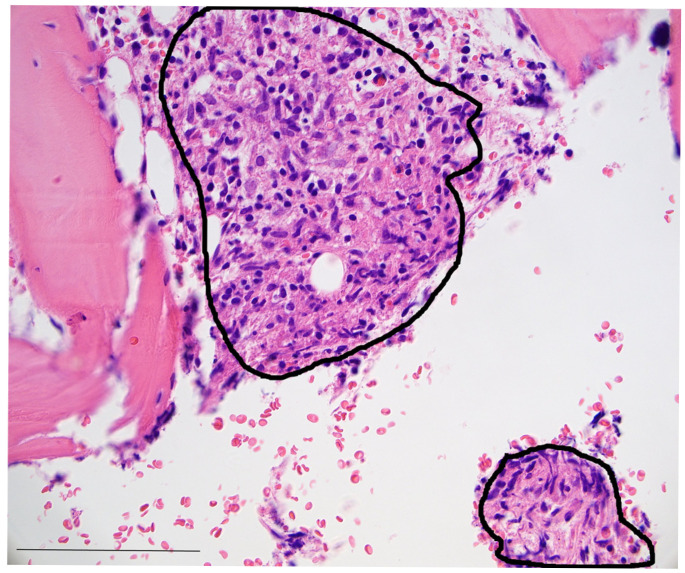
Photomicrography of the patient’s bone marrow biopsy (H&E stain, 400× magnification) showing loosely formed epithelioid granulomas (black encirclings) between bone trabeculae. Bar: 250 µm.

Bone marrow biopsy and aspiration were performed to further investigate pancytopenia and exclude myelodysplastic syndrome and acute myeloid leukemia. Histopathological workup of the performed bone marrow biopsy showed loosely formed epithelioid granulomas (see Figure 1), while cytological FACS analysis did not produce further insights, and microbiological testing initially remained negative. Soluble IL-2 receptor and ACE testing were performed and documented significant increases in both (ACE 137 U/L [normal 8–52 U/L]; IL-2-R 3252 U/mL [normal 158–623 U/mL]). Considering these histopathological and laboratory results, as well as radiological findings, sarcoidosis was diagnosed, and in the presence of bone marrow failure, prednisolone therapy was escalated to 1 mg/kg/day after the exclusion of infection with *M. tuberculosis* via QuantiFERON testing or leptospirosis. A dermatological examination yielded no indication for skin manifestations. A bronchoscopy to secure the diagnosis of sarcoidosis by lymph node biopsy was declined by the patient. Showing a significant overall clinical improvement, the patient was discharged.

Re-admission to the patient’s local hospital occurred two days after discharge with recurrent fever and hypotension. Pancytopenia was aggravated (hemoglobin 9.1 g/dL [14–17.5 g/dL], leucocytes 1.79 × 10^3^/µL [4–10 × 10^3^/µL], thrombocytes 43 × 10^3^/µL [140–450 × 10^3^/µL]), and CRP (13.2 mg/dL [<0.5 mg/dL]), and procalcitonin (0.58 ng/mL [<0.5 ng/mL]) was elevated. A hospital-acquired infection was suspected, and as no focus could be identified, therapy with meropenem and cotrimoxazole was initiated to include possible *Pneumocystis jirovecii* pneumonia. The hypotension required catecholamine therapy, and pancytopenia responded well to G-CSF injection, with the leucocytes normalizing completely. After initial stabilization, the patient was again referred to our tertiary center. Throughout observation, no episode of fever occurred. Extended serological diagnostics for histoplasmosis, aspergillosis, cryptococcosis, tuberculosis, syphilis, *Borrelia burgdorferi*, and *Francisella tularensis* remained negative. The patient was again discharged in stable condition.

**Figure 2:**
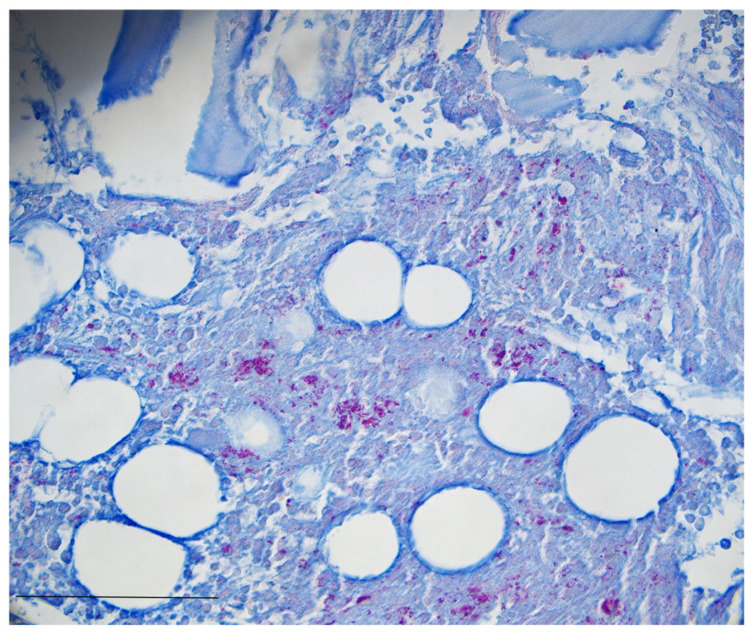
Ziehl–Neelsen stain reveals numerous acid-fast bacteria (Ziehl–Neelsen stain, 400× magnification) between bone trabeculae. Bar: 250 µm.

Two and a half weeks later, the patient was again admitted to secondary care with leading symptoms of weakness and fever. The attending primary care physician had initiated cefpodoxime and azithromycin therapy the day leading up to admission. Therapy quickly escalated to piperacillin/tazobactam, and pancytopenia was treated with G-CSF injection and blood transfusion. One day after re-admission and 6 weeks after initial bone marrow aspiration, *Mycobacterium genavense* infection was diagnosed. Bone marrow aspirate was inoculated in a BACTEC MycoF-Lytic blood culture in which acid-fast bacilli were seen using a Kinyoun stain. Ziehl–Neelsen staining of the initial bone marrow biopsy revealed acid-fast bacteria (see Figure 2), and *M. genavense* was identified using 16s rRNA gene sequencing. The identification was confirmed by the German National Reference Center for Mycobacteria, Borstel. Although the organism was isolated from multiple samples, subsequently, it could not be grown for antimicrobial susceptibility testing. For the initiation of therapy, the patient was transferred to our tertiary center.

Therapy was initiated in accordance with the recently published international consensus paper [17] with azithromycin at 500 mg, rifampicin at 600 mg, and ethambutol at 2000 mg per day, not requiring adjustment for renal function. Therapy with gyrase inhibitors was withheld due to contraindications, including coronary artery disease and aortic aneurysm, both present in the patient described. Additionally, treatment with aminoglycosides was avoided due to the need for daily intravenous application over a period of at least 12 months. In parallel, the prednisolone therapy for initially suspected sarcoidosis was reduced.

Two days after the initiation of adequate treatment, the leukocytes dropped from 4.73 × 10^3^/µL to 0.19 × 10^3^/µL, CRP increased to a maximum of 18.1 mg/dL, and the patient developed a significant hypotension, requiring high-dose norepinephrine therapy. Retrospectively, the episode was classified as immune reconstitution inflammatory syndrome (IRIS), as no failure of treatment, alternative infection, or drug side effect could be identified. The pancytopenia improved subsequently after additional blood and platelet transfusions were performed. Bilirubin, as well as coagulation, normalized. Due to persisting fever episodes with suspicion of activity of rheumatoid arthritis, methotrexate therapy was reinitiated in a reduced dosage. The fever episodes subsided four weeks into therapy with negative diagnostic testing, including blood cultures, bronchoscopy (BAL and EBUS-TBNA), transesophageal endoscopy, thoracic CT, and abdominal sonography, throughout the observation.

The fever recurred three months into therapy. With no additional focus being apparent and differential diagnostics remaining negative, therapy was continued without alteration. Regular follow-up visits every three months showed continuous improvements in the overall wellbeing of our patient. Methotrexate therapy was discontinued due to possible interference with a successful antimycobacterial treatment. Ten months into therapy, the patient developed increasing nausea and vomiting, leading to early termination of the treatment. As the inflammatory markers remained stable and the patient developed no further febrile episodes within six months after the end of treatment, NTM therapy was considered successfully concluded.

## 4. Discussion

Herein, we report the clinical manifestation of a disseminated nontuberculous mycobacterial infection with *M. genavense* of the bone marrow initially diagnosed as sarcoidosis under mild immunosuppressive therapy with methotrexate.

The case presented as fever of unknown origin (FUO), which is defined as body temperatures above 38.3 °C on at least two occasions and a duration of illness of at least three weeks, in spite of investigations on three outpatient visits or three days of stay in the hospital [18]. FUO presents a considerable challenge for clinicians given the very broad diagnostic workup.

NTM are ubiquitous, with more than 175 identified species and increasing numbers of infections [19]. Although NTM infections, and especially extrapulmonary manifestations, still must be considered rare, epidemiological studies have shown increasing prevalence. Different species are associated with different manifestations, such as *M. scrofulaceum* with cervical lymphadenitis in children, *M. xenopi* with osteoarticular infections, and *M. marinum* with soft tissue infections [20]. *M. genavense* was firstly described in 1992 in 18 patients with advanced HIV infection and disseminated disease by Böttger et al. [21]. The most common clinical presentation in immunocompromised patients, such as in the presented case, is disseminated disease [22,23,24]. Bowel and pleuropulmonary involvement are frequently described, while cutaneous, cerebral, and genital manifestations are less common. Case reports on immunocompetent patients are extremely rare. Interestingly, *M. genavense* is one of the most frequently isolated NTM from pet birds in Germany, presenting a possible reservoir of infection in the presented case [25,26]. Due to higher frequencies of abdominal manifestation in *M. genavense* compared to MAC infection, entrance of the pathogen through the gastrointestinal tract has been hypothesized [27].

Pathology findings from samples and biopsies are unspecific yet may direct us towards NTM infection, showing inflammatory changes, granulomas, and large amounts of intracytoplasmic acid-fast bacilli [28]. The findings of mediastinal and bilateral hilar lymphadenopathy, non-caseating granulomas of the bone marrow, and increased soluble IL-2 receptor and ACE lead to initial misdiagnosis and treatment as sarcoidosis, which highlights the diagnostic difficulty posed by disseminated *M. genavense* infections. Cases with similar misdiagnoses regarding *M. genavense* infection have been reported previously [29,30]. As differentiating between sarcoidosis and sarcoid-like lesions secondary to infection may be challenging, molecular techniques such as 16s rRNA gene sequencing are necessary to achieve and confirm diagnosis. Yet, *M. genavense* is not usually included in PCR panels used for the detection of mycobacteria in clinical routine, and Ziehl–Neelsen staining for acid-fast bacilli is limited by a detection limit of approximately 10^4^ organisms per milliliter, providing a significant hurdle in a slow-growing pathogen with low concentration, such as *M. genavense* [31].

Rapid and efficient identification of NTM is of clinical importance, as culture-based methods may take up to eight weeks to identify the relevant pathogens [32]. Although not yet in standardized clinical use, multiplex polymerase chain reaction assays, PCR restriction enzyme pattern analysis, or automated cycle sequencing of hypervariable region A of the gene encoding 16s rRNA may provide quick and reliable results in NTM testing [33,34,35]. Additional biological markers of NTM disease, such as bacterial cell wall products, immunoglobins, and gene expression of leucocytes in circulation, have been proposed to identify NTM disease [36]. Although these do not provide species identification, the sensitivity is partially reported to be higher than in cultures. Therefore, despite a considerable increase in knowledge regarding NTM infections, they still represent a diagnostic and therapeutic challenge. Pathogenic isolates are difficult to distinguish, timely identification of isolates is still limited, a lack of and limits to standardized susceptibility testing make targeted antibiotic therapy currently unrealistic, and a consequent lack of guidelines may result in suboptimal therapy.

Treatment regimens for *M. genavense* usually consist of at least three different antimicrobial agents, including macrolides, rifampicin (or rifabutin), and ethambutol [17]. Susceptibility to rifampicin and streptomycin, isoniazid resistance, and varying results regarding ethambutol are described in the literature. Additional options include gyrase inhibitors and aminoglycosides, each presenting limitations in the path of application or side effects. The temporary response to meropenem and cotrimoxazole described in the presented case therefore must be interpreted as non-specific. A recently published international consensus paper for less common NTM pulmonary diseases reiterates that the correlation of antimycobacterial drug susceptibility testing and clinical outcome can be poor, especially in *M. genavense* [17]. Additionally, resistance testing of the fastidious *M. genavense* is complicated, as the culture necessitates a liquid medium, acidic pH, and extended incubation due to its slow replication [16,37,38]. Yet, pharmacological treatment may be avoided in selected cases with debridement surgery as the source control [39]. In case reports, immunocompetence is described as a factor favoring this approach in patients with a risk of considerable side effects or lack of therapeutic options due to drug resistances [40].

IRIS as a paradoxical worsening of symptoms in NTM infections after starting adequate antimicrobial treatment is a well-documented phenomenon in both HIV and non-HIV patients following immunosuppression withdrawal. Differentiating IRIS from treatment failure can be challenging, as biomarkers such as IL-6 and CRP are unspecific, and rapid symptom resolution may support the diagnosis. A similar timeframe as in our case presentation has been described in a previous publication [41].

Our case report allows for the clear discrimination of *M. genavense* infection from sarcoidosis, as the patient likely acquired the infection under the immunosuppressive therapy for rheumatoid arthritis and presented worsening symptoms under higher doses of prednisolone before starting adequate antimicrobial therapy. Indeed, the follow-up with IRIS and complete resolution of febrile episodes and cytopenia support this view.

In patients with a fever of unknown origin and suspicion of sarcoidosis, NTM infections should always be considered as a differential diagnosis, and mycobacterial diagnostics should be performed. Yet, the outcomes remain unfavorable even within the more common pulmonary NTM infections, with mortality rates ranging from 29 to 69% despite developments in resistance testing and antibiotics [42,43,44]. Recently published consensus guidelines on pulmonary NTM infections present a first step in the direction of evidence-based treatment of NTM infections, including rare specimens such as *M. genavense*. Further progress in the rapid identification of NTM is needed, as delays of therapy may result in unfavorable patient outcomes.

## Figures and Tables

**Table 1:** Initial patients characteristics and laboratory results.

Baseline Characteristics			Reference Values
Age	73 years		
Gender	Male		
Occupation	Pensioner, pigeon fancier		
Symptoms	Reoccurring fever, pronounced weakness, pancytopenia, reduced vigilance		
Medical history	BMI 31.2 kg/m², rheumatoid arthritis, myocardial infarction, aortic aneurysm, cholecystolithiasis, diverticulosis, splenomegaly, spondylodesis		
Family history	None		
Medication	Acetylsalicylic acid Prasugrel Bisoprolol Doxycycline Folic acid Methotrexate Leflunomide Prednisolone Oxycodone Pantoprazole Rosuvastatin Spironolactone Tamsulosin	100 mg/day 10 mg/day 2.5 mg/day 200 mg/day 5 mg/day Paused, 20 mg/week 20 mg/day 20 mg/day 40 mg/day 40 mg/day 20 mg/day 25 mg/day 0.4 mg/day	
Initial laboratory results			
Leucocytes	2.05 × 10^3^/µL		[3.91–10.9 × 10^3^/µL]
Neutrophils	81.8%		41–70.7%
Lymphocytes	11.1%		19.1–47.9%
Monocytes	5.2%		5.2–15.2%
Eosinophils	1.2%		0.6–7.6%
Basophils	0.6%		0.1–1.2%
Hemoglobin	8.1 g/dL		[13.5–16.9 g/dL]
Thrombocytes	101 × 10^3^/µL		[166–308 × 10^3^/µL
Bilirubin	1.6 mg/dL		[<1.2 mg/dL]
Serum creatinine	1.22 mg/dL		[<0.9 mg/dL]
C-reactive protein	3.2 mg/dL		[<0.5 mg/dL]
Initial blood pressure	95/66 mmHg		

## Data Availability

The data presented in this study are available upon reasonable request and with permission of the patient from the corresponding author due to privacy policies.
